# Supplementary material for: No evidence for carcinogenicity of titanium dioxide nanoparticles in 26-week inhalation study in rasH2 mouse model
Source: Sci Rep. 2022 Sep 2;12:14969. doi: 10.1038/s41598-022-19139-y (PMC9440215; doi:10.1038/s41598-022-19139-y)
Supplement: Supplementary file 1 — Supplementary Figures. [file 41598_2022_19139_MOESM1_ESM.pdf]

Fig. S1

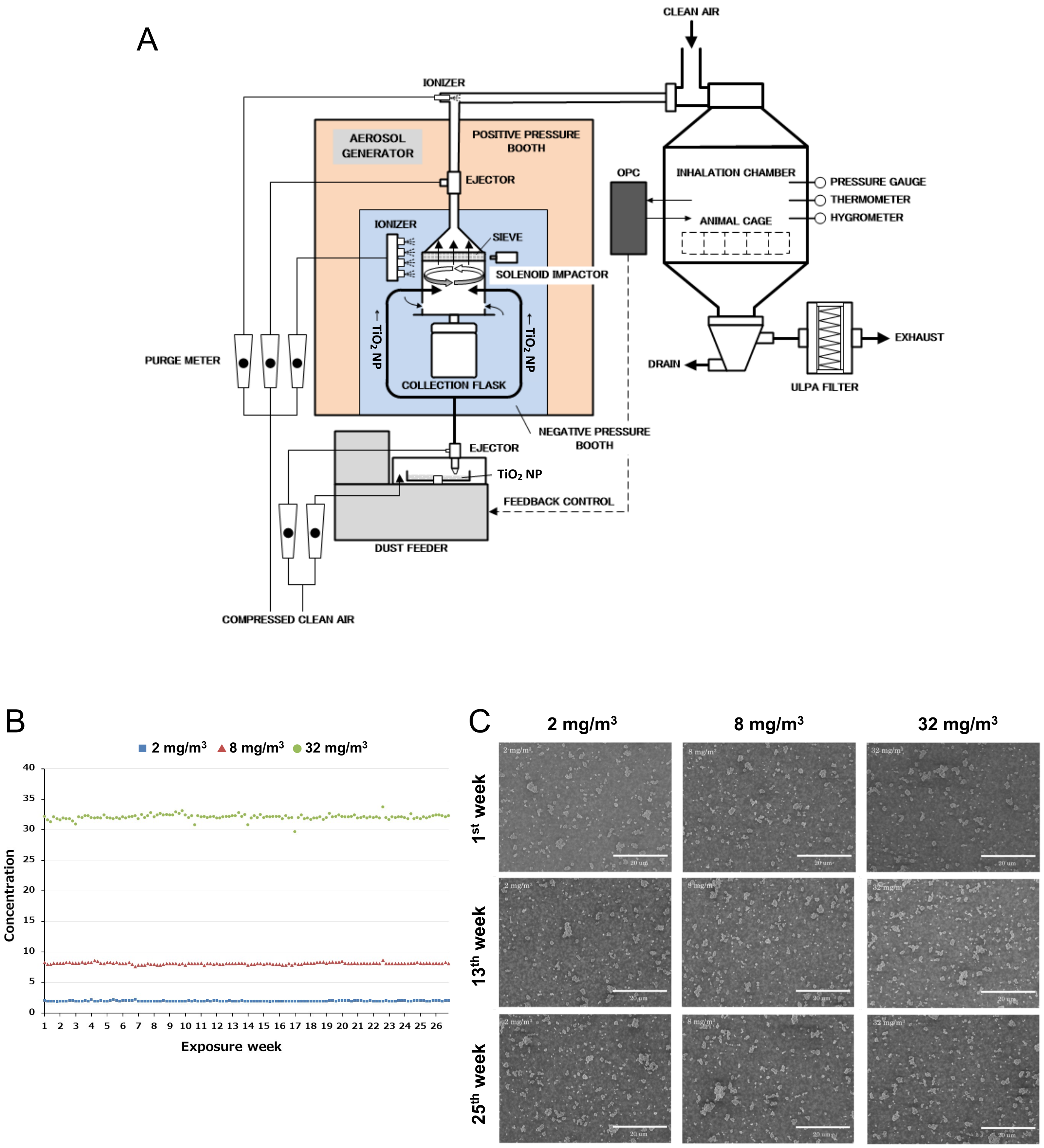

A
CLEAN AIR
IONIZER
AEROSOL GENERATOR
POSITIVE PRESSURE BOOTH
EJECTOR
OPC
INHALATION CHAMBER
PRESSURE GAUGE
THERMOMETER
HYGROMETER
ANIMAL CAGE
IONIZER
SIEVE
SOLENOID IMPACTOR
TiO2 NP
TiO2 NP
PURGE METER
COLLECTION FLASK
DRAIN
EXHAUST
ULPA FILTER
NEGATIVE PRESSURE BOOTH
EJECTOR
TiO2 NP
FEEDBACK CONTROL
DUST FEEDER
COMPRESSED CLEAN AIR
B
2 mg/m3 8 mg/m3 32 mg/m3
Concentration
0 5 10 15 20 25 30 35 40
1 2 3 4 5 6 7 8 9 10 11 12 13 14 15 16 17 18 19 20 21 22 23 24 25 26
Exposure week
C
2 mg/m3
8 mg/m3
32 mg/m3
1st week
13th week
25th week

Fig. S1

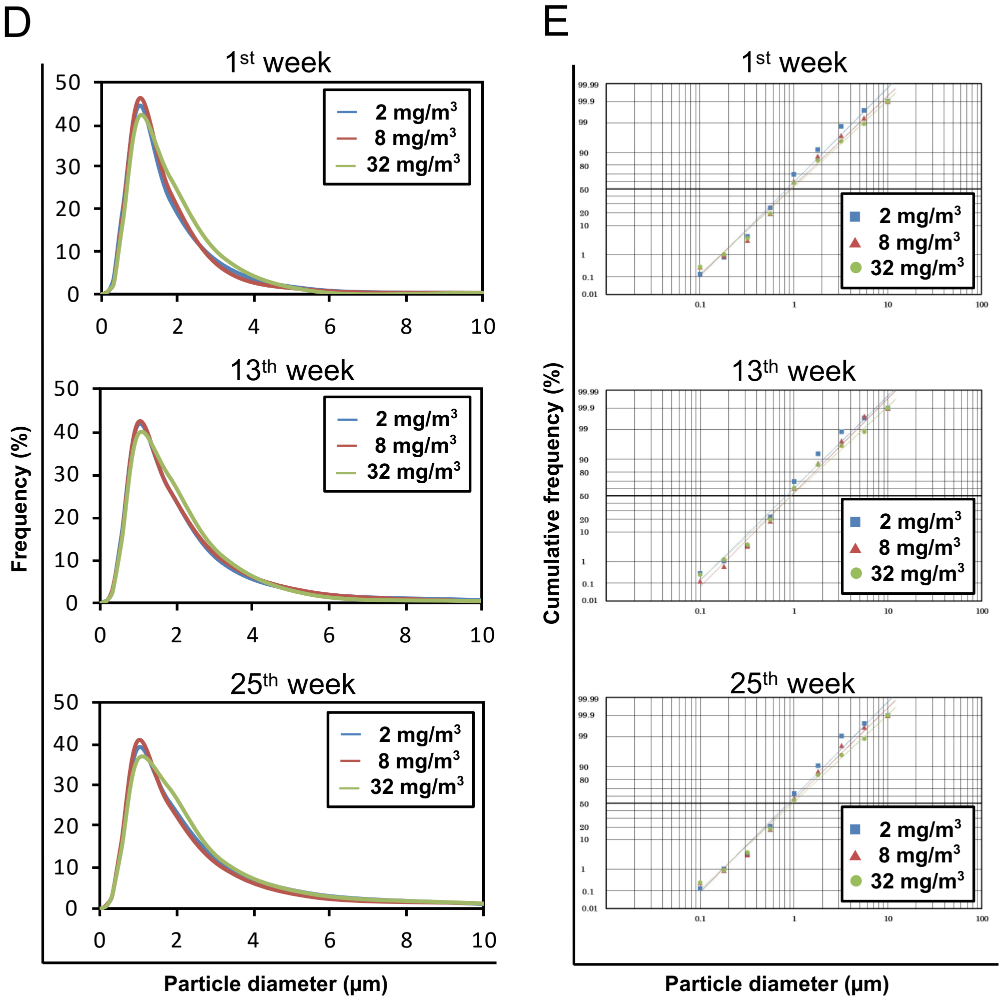

F

Exposure Concentration	1st exposure		13th exposure		25th exposure	
	MMAD (μm)	σg	MMAD (μm)	σg	MMAD (μm)	σg
2 mg/m^3	0.8	2.0	0.9	2.1	1.0	2.1
8 mg/m^3	0.8	2.0	0.9	2.0	0.9	2.1
32 mg/m^3	0.9	2.0	0.9	2.0	1.0	2.1

Fig. S1

The whole body inhalation exposure system in this study.

The whole body inhalation exposure system (A), the averaged TiO_2 NPs concentration in the chamber per each exposure day (B), representative scanning electron microscope (SEM) images of the TiO_2 NPs particles in the chambers (C), particle-size distribution of the TiO_2 NPs particles in the chambers (D), cumulative frequency distribution graphs with logarithmic probability (E) and Mass median aerodynamic diameter (MMAD) and geometric standard deviation (σg) in the chamber (F) and. Scale bar: 20 μm (panel E).

Fig. S2

Experimental protocol
(inhalation study: dose-response)

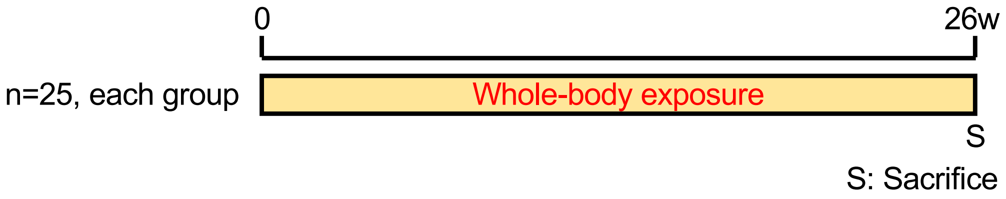

Animal: Jic:CB6F1-Tg rasH2@Jcl (rasH2) mice, 8-week-old
male (n=25 each group), female (n=25 each group), total n=200
Test compounds: Anatase type, titanium dioxide nanoparticle (primary particle size: 30 nm)
Exp. Conc.: 6hr/day, 5 day/week, 0, 2, 8, and 32 mg/m^3

Fig. S2
Design of animal experimental protocol used for this study.

Fig. S3

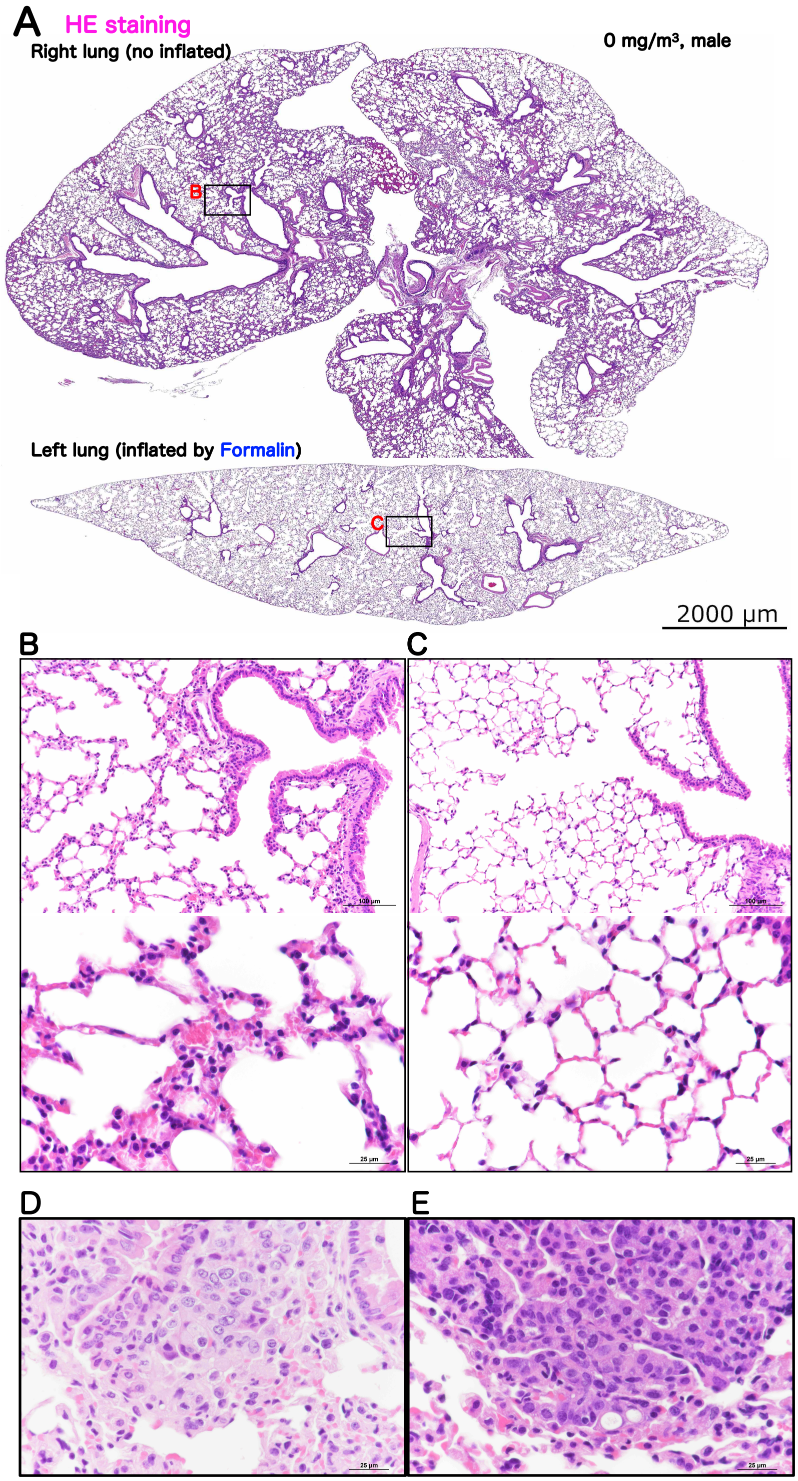

Fig. S3

Representative microscopic photographs of lungs of a male control rasH2 mouse.

The lungs were stained with hematoxylin and eosin (HE) (see the Fig. 3 legend for details). A typical loupe image (A) of the entire lungs and magnified images of normal alveolar regions (B and C) have been shown. Bronchiolo-alveolar adenoma of a male control rasH2 mouse in this study (D) and our historical control data (E).

Fig. S4

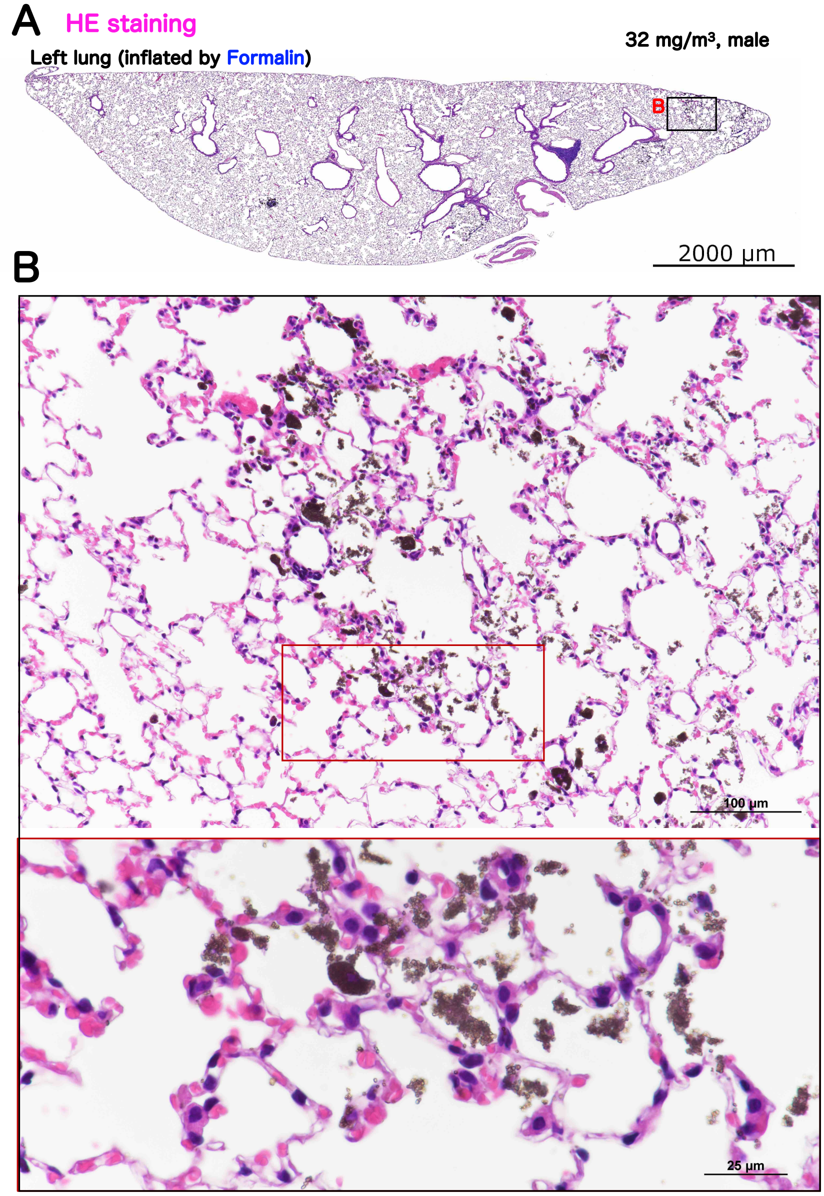

Fig. S4

Representative microscopic photographs of male rasH2 mouse left lung after inhalation exposure to TiO_2 NPs (32 mg/m^3), same mouse of fig.2.

For the left lung, formalin was injected into the lung through the bronchus and stained with HE. A typical loupe image (A) of the entire right lungs and magnified image of inflammatory focus (B) have been shown. The inflammatory foci observed in the right lung were scattered in the left lung due to the injection of formalin, making it difficult to observe them clearly.

Fig. S5

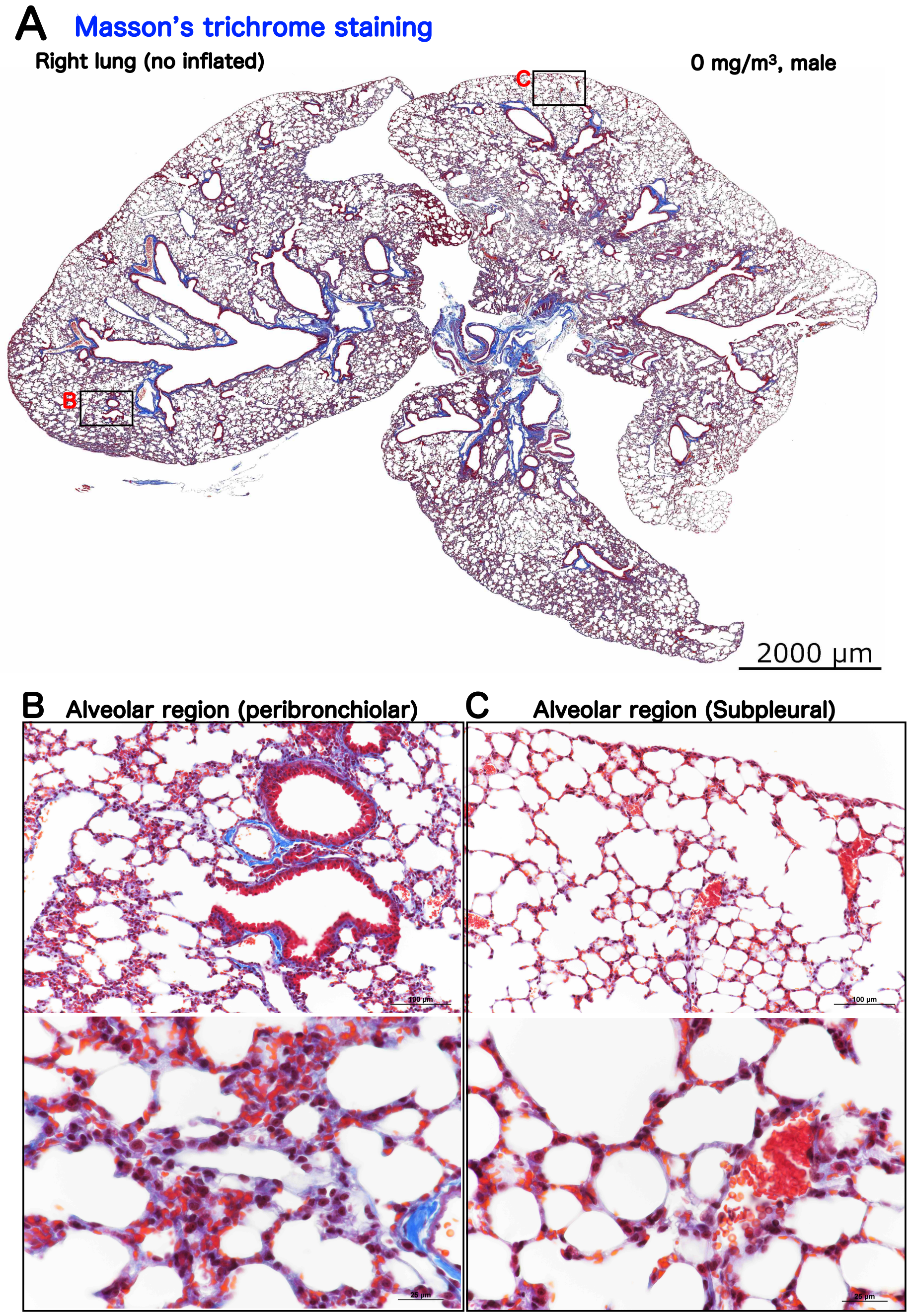

Fig. S5

Representative microscopic photographs of Masson's trichrome staining of male control rasH2 mouse right lungs.

A typical loupe image (A) of the entire right lungs and magnified images of peribronchiolar (B) and subpleural (C) alveolar regions have been shown.
